# Growing Trend to Adopt Speckle Variance Optical Coherence Tomography for Biological Tissue Assessments in Pre-Clinical Applications

**DOI:** 10.3390/mi15050564

**Published:** 2024-04-25

**Authors:** Ruchire Eranga Wijesinghe, Nipun Shantha Kahatapitiya, Changho Lee, Sangyeob Han, Shinheon Kim, Sm Abu Saleah, Daewoon Seong, Bhagya Nathali Silva, Udaya Wijenayake, Naresh Kumar Ravichandran, Mansik Jeon, Jeehyun Kim

**Affiliations:** 1 Department of Electrical and Electronic Engineering, Faculty of Engineering, Sri Lanka Institute of Information Technology, Malabe 10115, Sri Lanka; eranga.w@sliit.lk; 2 Center for Excellence in Intelligent Informatics, Electronics & Transmission (CIET), Sri Lanka Institute of Information Technology, Malabe 10115, Sri Lanka; 3 Department of Computer Engineering, Faculty of Engineering, University of Sri Jayewardenepura, Nugegoda 10250, Sri Lanka; egt18538@sjp.ac.lk (N.S.K.); udayaw@sjp.ac.lk (U.W.); 4 Department of Artificial Intelligence Convergence, Chonnam National University, Gwangju 61186, Republic of Korea; 5 Department of Nuclear Medicine, Chonnam National University Medical School & Hwasun Hospital, 264, Seoyang-ro, Hwasun 58128, Republic of Korea; 6 ICT Convergence Research Center, Kyungpook National University, 80, Daehak-ro, Buk-gu, Daegu 41566, Republic of Korea; 7 School of Electronic and Electrical Engineering, College of IT Engineering, Kyungpook National University, 80, Daehak-ro, Buk-gu, Daegu 41566, Republic of Korea; 8 Faculty of Computing, Sri Lanka Institute of Information Technology, Malabe 10115, Sri Lanka; 9 Center for Scientific Instrumentation, Korea Basic Science Institute, 169-148, Gwahak-ro, Yuseong-gu, Daejeon 34133, Republic of Korea

**Keywords:** speckle-variance optical coherence tomography (SV-OCT), biological tissue imaging, microvasculature mapping, pre-clinical monitoring, blood flow assessment, *in vivo* vascular assessment

## Abstract

Speckle patterns are a generic feature in coherent imaging techniques like optical coherence tomography (OCT). Although speckles are granular like noise texture, which degrades the image, they carry information that can be benefited by processing and thereby furnishing crucial information of sample structures, which can serve to provide significant important structural details of samples in *in vivo* longitudinal pre-clinical monitoring and assessments. Since the motions of tissue molecules are indicated through speckle patterns, speckle variance OCT (SV-OCT) can be well-utilized for quantitative assessments of speckle variance (SV) in biological tissues. SV-OCT has been acknowledged as a promising method for mapping microvasculature in transverse-directional blood vessels with high resolution in micrometers in both the transverse and depth directions. The fundamental scope of this article reviews the state-of-the-art and clinical benefits of SV-OCT to assess biological tissues for pre-clinical applications. In particular, focus on precise quantifications of *in vivo* vascular response, therapy assessments, and real-time temporal vascular effects of SV-OCT are primarily emphasized. Finally, SV-OCT-incorporating pre-clinical techniques with high potential are presented for future biomedical applications.

## 1. Introduction

**Figure 1:**
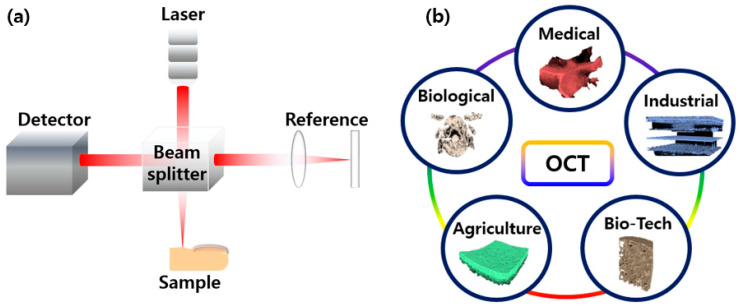
(**a**) An overview of the optical coherence tomography (OCT) system schematic and (**b**) the extensive distribution of applications.

Optical coherence tomography (OCT) is an optical non-destructive imaging modality that is capable of providing cross-sectional and three-dimensional (3D) images [1,2] with micrometer resolutions. OCT offers a high level of sensitivity for depth-resolved images with a high signal-to-noise ratio at a depth range of 2–8 mm [3,4]. OCT was first demonstrated in 1991 for retinal imaging [1]. OCT is an interferometric imaging technique that uses near-infrared (NIR) light to map sub-surface reflections to generate cross-sectional images with detailed morphological features [5,6,7] with intensity variations with respect to the varying refractive index of microstructures within the sample. The advent of Fourier domain OCT (FD-OCT) and its high sensitivity facilitated the progression from real-time two-dimensional (2D) imaging to real-time volumetric imaging [8,9], with the maximum imaging depth and field of view varying based on the system specifications [10,11,12]. An overview of the OCT system schematic together with the extensive distribution of applications are presented in Figure 1a,b.

OCT can be sub-categorized by functional methods depending on the different properties of light, namely, optical Doppler tomography (ODT), phase-sensitive OCT (Phs-OCT), phase variance OCT, polarization-sensitive OCT (PS-OCT), photothermal OCT (PT-OCT), and speckle variance OCT. The interest in developing contrast agents for targeting and functioning as molecular contrast agents in OCT, ODT, and PT-OCT has recently become notable. Enhanced *in vivo* contrast within a sample can be achieved through the notable scattering efficiency of contrast agents [13,14,15]. The information determined from the polarization state of the interference fringe in the detected OCT signals helps in developing PS-OCT, which is a valid functional extension of OCT to obtain an additional contrast in OCT images [16,17]. PS-OCT is a valuable tool to obtain high-resolution spatial information on the polarization state of the reflected light from the microstructures of the specimen.

**Figure 2:**
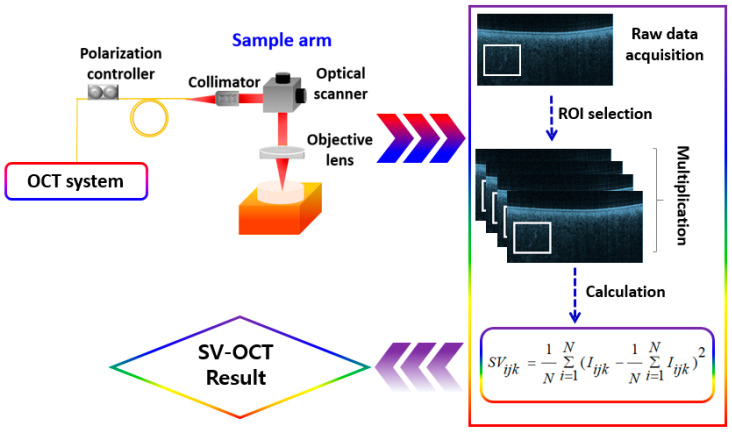
The representative schematic of the SV-OCT system.

Speckle variance optical coherence tomography (SV-OCT) is a technique used in biomedical imaging, particularly in ophthalmology, to visualize microstructural information within tissues. SV-OCT is a functional extension of OCT, which has recently gained enormous interest as a trending micro-angiography technique. SV-OCT is based on conventional OCT, which utilizes low-coherence interferometry to obtain cross-sectional images of tissue microstructure. Speckle patterns arise due to interference from backscattered light in the sample. In OCT images, these speckle patterns contain information about the tissue microstructure. Speckle patterns change over time due to movement or changes within the sample. This dynamic nature is utilized in SV-OCT to extract additional information about the sample’s internal structures, which is unavailable in traditional OCT images. SV-OCT images are obtained by calculating the variance of speckle intensity within a series of OCT images acquired over a short period. In the calculated signals, the regions with high variance indicate areas of movement or change within the sample. This label-free contrast-enhanced imaging technology can be utilized to map microvasculature structures, yielding depth-resolved visualizations of micrometer-resolved blood vessels with exceptional contrast [17]. Further, in this technology, moving molecules in microvasculature structures lead to generating alterations of speckle patterns, which can be quantitatively acquired by estimating speckle variances between frames or lines. The initial use of speckle analysis in OCT images to obtain depth-resolved blood flow was first documented in 2005 [18]. The speckle variance (SV) algorithm was applied to visualize blood vessels [19,20,21]. The SV algorithmic calculation used in SV-OCT involves several steps for processing the acquired OCT B-scans and extracting speckle variance information. First, a stack of consecutive B-scans is obtained rapidly, typically using a high-speed OCT imaging system. Within this stack, a region of interest (ROI) is selected for analysis, which may correspond to a specific tissue area or depth within the sample. Next, for each pixel position within the ROI, the intensity values across the selected frames are extracted. Then, the variance of these intensity values is computed, typically using the standard deviation calculation, to quantify the level of speckle variance at each pixel. To enhance visualization and facilitate comparison between different samples or regions, the variance values may be normalized. Finally, the resulting speckle variance image is generated, where areas of high variance correspond to regions of movement or change within the sample. This algorithm enables the visualization and analysis of dynamic processes, such as blood flow or tissue motion, providing valuable insights into biological structures and functions. Owing to these diverse functional capabilities, SV-OCT gained the spotlight as a potential imaging tool for microvasculature in biological specimens. While 2D cross-sectional images of SV-OCT provide depth-resolved *in vivo* images of biological specimens, the 3D vascular images are acquired by calculating the inter-frame variance. The schematic of Figure 2 depicts the widely adopted detection algorithm of the SV-OCT system [22,23], and the system configurations of SV-OCT systems can be found in [24]. A high-configuration personal computer was assembled using a high-performance GPU to obtain continuous real-time acquisition, fast image processing, and to display the structural and SV calculations in real-time. The accuracy of SV-OCT measurements is affected by the SV contrast between biological microstructures and their nearby fluid components. Further technical aspects of SV-OCT are illustrated descriptively elsewhere [25,26]. 

The purpose of this article is to review the state-of-the-art and pre-clinical benefits of label-free SV-OCT for assessing quantifications of *in vivo* vascular response, therapy assessments, and real-time temporal vascular effects as an imaging method with enhanced contrast. The article begins with a technical discussion and a contrasted comparison between pre-clinical translation and quantification capabilities of SV-OCT for biological tissue assessment to lay the foundation for understanding its strengths and limitations. To understand the best applicability of this method, a further comparison of therapeutic assessments of various medical treatments is provided. The review concludes with a summarized discussion illustrating the key features and strengths of previously reported SV-OCT studies in biomedical applications, as well as highlighting the most expected future trends.

## 2. The Pre-Clinical Translation and Quantification Capabilities of SV-OCT for Biological Tissue Assessment

**Figure 3:**
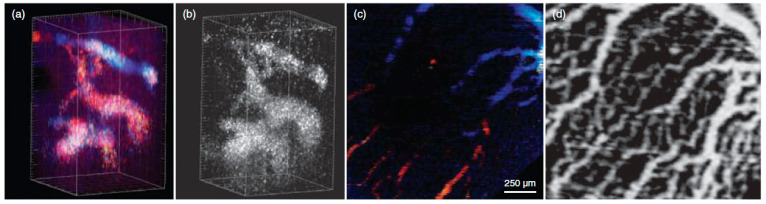
SV OCT and Doppler OCT visualization of embryonic vasculature. (**a**) Doppler OCT provides a 3D reconstruction of the vasculature in the mouse embryonic brain at 8.5 dpc; (**b**) Corresponding reconstruction acquired through SV analysis, revealing the same identified vascular structures; (**c**) Doppler OCT generates a 3D reconstruction of the yolk sac vasculature at 9.5 dpc; (**d**) SV analysis-acquired corresponding reconstruction of the yolk sac vasculature, illustrating a more complex and well-defined vascular structure. The major ticks in (**a**,**b**) correspond to 50 μm (adapted from [30]).

Abnormalities in blood flow can lead to cardiological and vasculature defects [27,28,29], as appropriate blood flow is essential for the development of the heart and other organs. This involves diagnosing and assessing the vascular network and its health, along with measuring blood flow rates to assess the condition of organ health. In clinical and research settings, microscopes and endoscopes are predominantly used for the diagnosis of vascular health. However, the commonly adopted medical endoscopes offer only the surface of tissues, and this makes it difficult to estimate the underlying tissue health, which results in limiting the estimation of the overall condition of the vasculature. The use of SV-OCT not only enables 3D volumetric visualization of the vasculature but also enables the assessment of the sub-layer conditions. The effective use of SV-OCT with its 3D vasculature reconstruction of live mouse embryos was demonstrated in [30], and showed the advantages of SV-OCT to visualize blood vessels in transverse and in depth. The study results were obtained using both SV-OCT and Doppler OCT systems, as shown in Figure 3, and equal performance was observed in both systems when blood flow had a significant axial component. SV-OCT demonstrated better performance (Figure 3b,d) in visualizing the network and branching of vasculature compared to Doppler OCT (Figure 3a,c), making it beneficial for vasculature remodeling studies.

**Figure 4:**
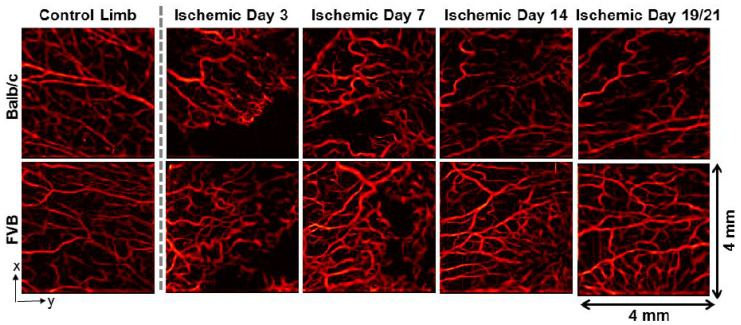
Representative SV-OCT images of the adductor muscle in Balb/c (**top row**) and FVB (**bottom row**) mice, including the contralateral control limb on the far left and the ischemic limb. Imaging was conducted non-invasively over time, with the final time point at day 19 for Balb/c mice and day 21 for FVB mice (adapted from [40]).

**Figure 5:**
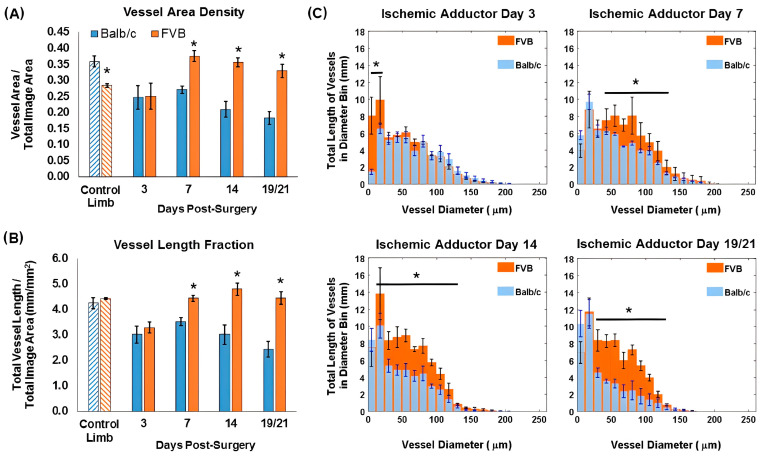
Assessment of vascular morphology metrics using SV-OCT projection images for Balb/c (n = 4) and FVB (n = 3) mice. FVB mice demonstrated increased (**A**) vessel area density and (**B**) vessel length fraction at day seven and subsequent post-surgery time points in the ischemic adductor region compared to Balb/c mice (* *p* < 0.05 between strains). Balb/c mice showed a decrease in both parameters between days 7 and 19 (*p* < 0.05), while FVB mice exhibited an increase in vessel area density and length fraction between days 3 and 7 and days 3 and 14, respectively (*p* < 0.05). (**C**) Notably, significant differences in the length of vasculature within a specified range of vessel diameters were also observed (* *p* < 0.05 for the indicated diameter range). The imaging concluded on day 19 for Balb/c mice and day 21 for FVB mice (adapted from [40]).

In particular, cancer, cardiovascular diseases, and the cells surrounding the scars aid cells in manipulating their microenvironment for remodeling [31,32,33]. This has given rise to a growing interest in animal model studies to understand vascular remodeling [34,35]. Due to the complexity of the vascular remodeling process, a non-invasive quantification of *in vivo* vascular remodeling, which can be studied over the entire remodeling process, serves to be highly beneficial. However, obtaining data with high accuracy and visualizing and understanding the overall ongoing process during the course of remodeling can be challenging with conventional histological assessments and other optical medical imaging methods [36,37,38]. Although conventional OCT provides qualitative information with high resolution, quantitative metrics of vascular morphology were not sufficient to assess the overall condition of vasculature in its entirety [39]. To overcome this, Poole et al. used SV-OCT to understand the mechanisms and dynamics of the vascular remodeling process for different pathological conditions, from ischemia to cancer [40]. In this demonstration, mouse models with robust (Friend Virus B-type—FVB) [41] and poor (Balb/C) recovery [42] to hind limb ischemia were used to acquire quantitative vascular SV-OCT images. Figure 4 presents SV-OCT images of the ischemia limb adductor from each mouse strain, as demonstrated in the Poole et al. study [40], confirming the visualization of vessel remodeling over time. The results emphasize that the average intensity projection of volumetric SV over 1.5 mm depth revealed notable variances in vascular response. The results provide a clear dimensional comparison between data acquired on each monitoring day. Figure 5 depicts the acquired SV-OCT quantitative information from two different mouse models. The promising capability of SV-OCT to assess various vascular responses was successfully confirmed through the results. 

**Figure 6:**
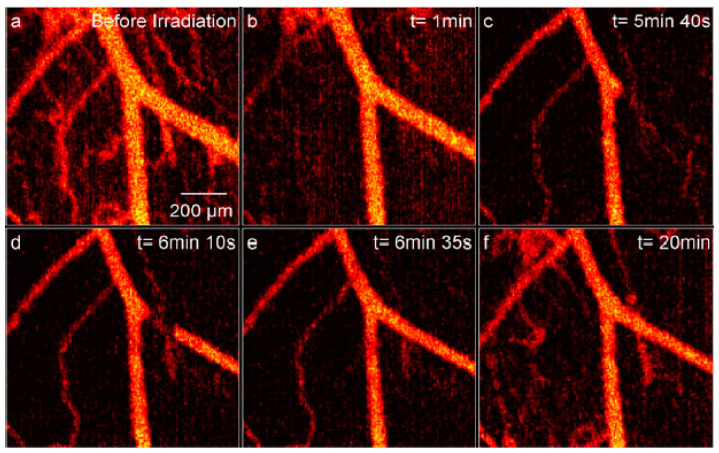
(Color online) SV-OCT records Visudyne-mediated PDT in a 1 mm × 1 mm region of the dorsal skinfold window chamber mouse model (fluence rate = 42 mW/cm^2^, total fluence = 25 J/cm^2^, treatment time = 10 min). (**a**) Presents vasculature before laser irradiation. (**b**) Depicts one minute after the start of laser irradiation. (**c**) Demonstrates the total shutdown of the right branch. (**d**) Shows the reperfusion of the right branch with an imaging artifact. (**e**) Illustrates the reperfusion of the right branch without imaging artifact. (**f**) Displays the condition 10 min post-end of laser irradiation, indicating reperfusion, but main vessels still appear constricted (adapted from [20]).

To further enhance the qualitative representation, Fourier domain mode-locking [43,44] provides efficient microvascular detection, as reported in [20,45,46]. This implementation enables the detection of vessels within the range of 0–25 μm through the maximum intensity projection of enface maps. As illustrated in Figure 6, real-time implementation and additional Doppler angle-independent microvascular information [47] are the main advantages of SV-OCT over conventional Doppler-OCT. Figure 6 illustrates the identification of microvascular changes induced by Visudyne photodynamic therapy (PDT) through imaging before (Figure 6a), during (Figure 6c–e), and immediately post-treatment (Figure 6f).

**Figure 7:**
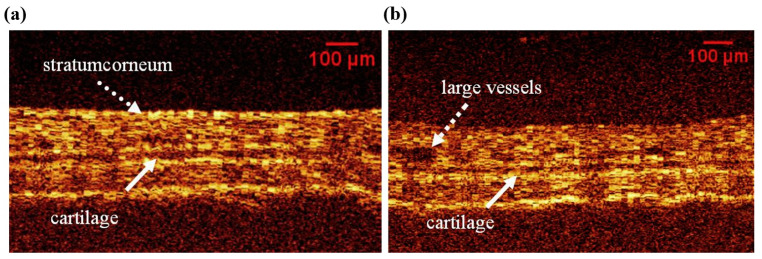
Representative OCT images of mouse ear. (**a**) Normal mouse ear. (**b**) Illustrates the ear after acetone-mediated exfoliating. Scale bar = 100 μm (adapted from [50]).

**Figure 8:**
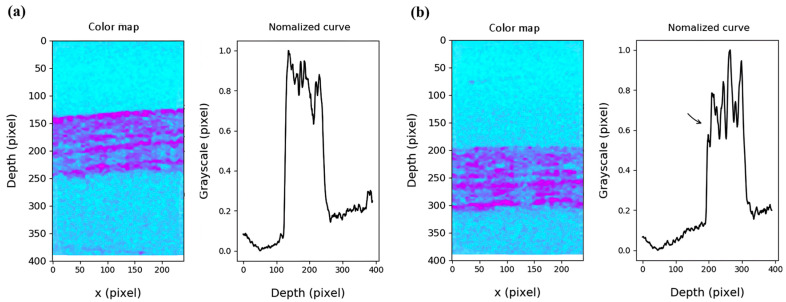
Color map and normalized curve of gray value in the longitudinal distribution. (**a**) Illustrates the condition before acetone treatment. (**b**) Shows the state after acetone treatment. The black arrow indicates the disappearance of the corneum peaks (adapted from [50]).

Cutaneous tissue swelling is another challenging skin disease that requires accurate quantification [48], where histological and weight measurements have been primarily applied for assessments [49]. The results from the Li, W et al. study, as shown in Figure 7a, demonstrated that the changes in OCT image grey values corresponded to the presence of histamine in sub-surface layers. Conversely, Figure 7b depicts the disappearance of the bright corneum line in the OCT image following pre-treatment with acetone, indicating the effect of acetone-induced exfoliating on the mouse ear skin. Moreover, the results shown in Figure 8 indicate the color map and normalized quantitative information of the difference value measurements acquired before (Figure 8a) and after exfoliation (Figure 8b). Although the obtained results exhibited potential benefits, the information was limited to a specific depth range and only to a small portion of the mouse ear, which are essential parameters for examining tissue swelling. 

**Figure 9:**
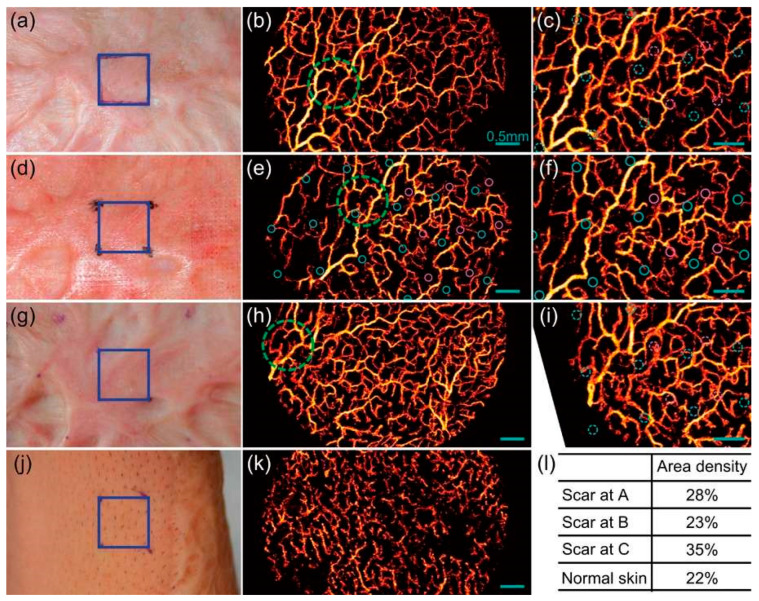
Imagine blood vessels within a mature scar (**a**–**i**) and adjacent normal skin (**j**,**k**). Photographs (**a**,**d**,**g**) alongside corresponding vasculature maximum intensity projections (MIPs) (**b**,**e**,**h**) depict central regions within the blue square outlines (10 mm × 10 mm) on the scar at time points A, B, and C. Noteworthy vessel patterns are highlighted by green dashed circles. Overlapping regions are shown in (**c**,**f**,**i**). A photograph of normal skin is presented in (**j**), with the corresponding vasculature MIP in (**k**). Vasculature area density in the scar at time points A, B, and C, along with normal skin, is indicated in (**l**). The small cyan and purple solid circles in (**e**,**f**) represent microthermal treatment zones (MTZs) from the first laser treatment, corresponding to two adjacent scan paths of the laser microscanner. After registration, their locations are illustrated as a guide in (**c**,**i**) using dashed circles. Scale bars: 0.5 mm. All vasculature MIPs are derived from the skin surface to a depth of 300 μm (adapted from [52]).

As mentioned above, investigating scar progression [51] is another SV-OCT application where OCT monitors the vasculature during wound healing. Though numerous optical techniques were performed to assess scar progression, SV-OCT was favored as one of the most feasible techniques. As a precise verification, P. Gong et al. [52] examined microvascular changes during wound healing of burn scars, which were treated using fractional laser [53]. Several patients were continuously monitored over a period using SV-OCT. The SV-OCT results shown in Figure 9 confirmed that the wounded or scar tissues (Figure 9a–i) have a higher degree of vasculature than normal tissues (Figure 9j,k). In contrast, a reduction of vasculature degree was observed along with the laser treatment.

**Figure 10:**
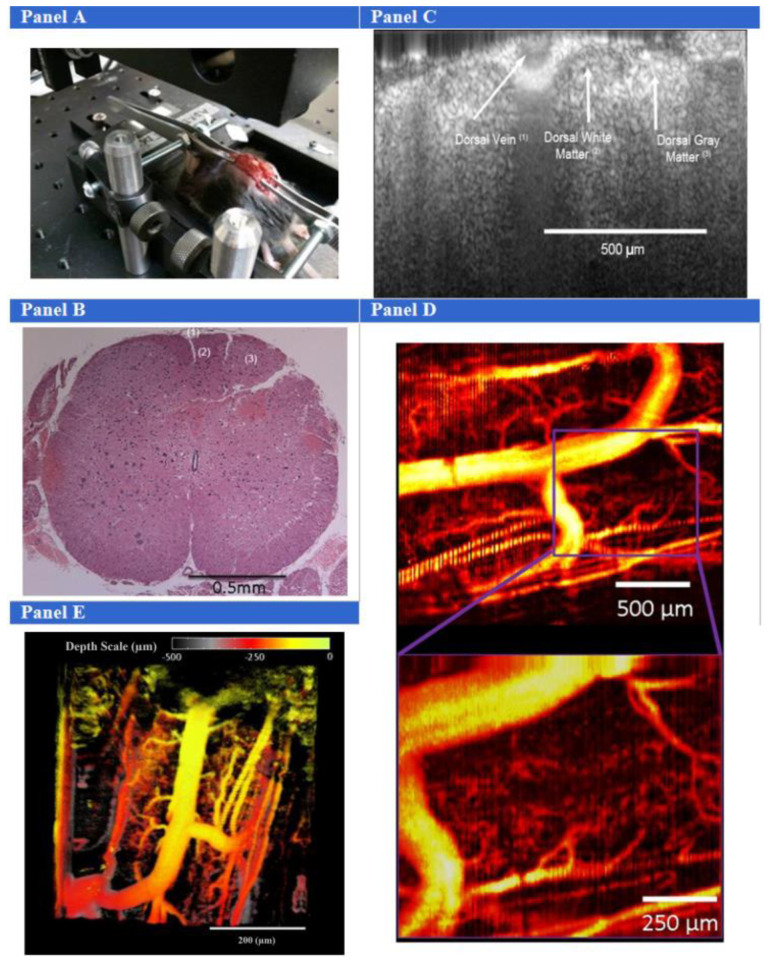
The experimental setup for mouse SV-OCT imaging included correcting the bulk motion of the spinal column using a custom jig, as depicted in (**Panel A**). This technique involved delicately grasping the spinal column with two pairs of forceps, positioned one vertebral body level above and below the exposed spinal cord. (**Panel B**) illustrates a histology specimen showing the dorsal vein (1), dorsal white matter (2), and dorsal gray matter (3). In (**Panel C**), a structural OCT image displays the dorsal vein and dorsal white and gray matter. (**Panel D**) showcases SV-OCT images revealing the intricate microvascular network of the mouse spinal cord, resolving vessels with a diameter of about 10–20 μm. Furthermore, (**Panel E**) presents a depth-dependent false color map of the mouse spinal cord (adapted from [22]).

In addition to cardiovascular assessments, high spatial and temporal resolutions of SV-OCT offer unique advantages for investigating rodent vasculature [32], which are beneficial for treating the spinal cord. The SV-OCT imaging technology can provide depth-resolved imaging of microvascular networks without limiting sensitivity to the Doppler angle. Also, it is yet to be discovered that the neurovascular signaling mechanisms underlying functional hyperemia in the spinal cord are similar to those in the cerebral cortex. The neurovascular coupling mechanisms differ across neuroanatomical pathways in the cerebral cortex and across brain regions. These mechanistic differences exist in the evolutionary older spinal cord, and remain to be determined, as does their effect on functional hyperemia. SV-OCT technology has performed as a promising tool in discovering this information in healthy spinal cord states and certain disease states. The results of the Cadotte, D.W. et al. study, as illustrated in Figure 10 encompassing panels A to E, demonstrate the SV-OCT information with a significant visualization of cardiorespiratory motion in the lumbar region of the mouse spinal cord [22]. Although the acquired results confirmed vital discoveries of the spinal cord, an optimized method is required to overcome the limitations of bulk motions.

**Figure 11:**
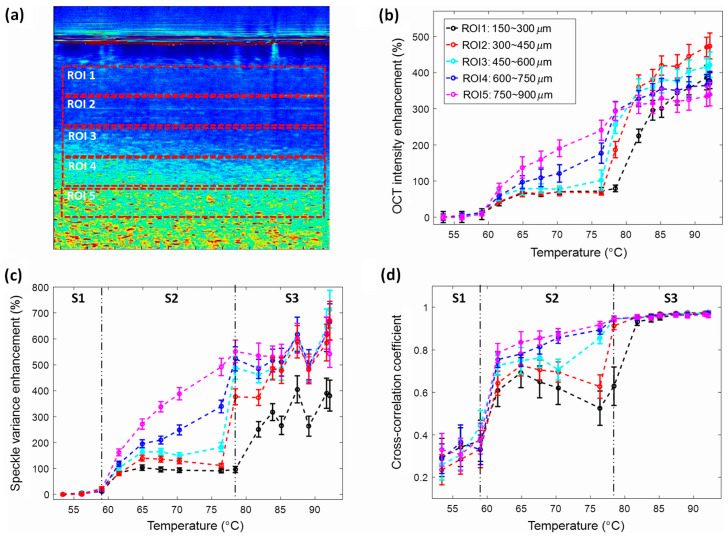
Evaluation of regions of interest (ROIs) at 16 heating temperatures. (**a**) Exhibits five chosen ROIs in the SV-OCT image at 76.4 °C. (**b**) Demonstrates OCT intensity enhancement, (**c**) illustrates SV enhancement, and (**d**) displays cross-correlation coefficients at these five selected ROIs over 16 heating temperatures. Abbreviations: S1, stage 1; S2, stage 2; and S3, stage 3 (adapted from [55]).

Thermal-induced protein denaturation and coagulation [54] occur in biological tissues by changes in temperature since the stability of the 3D protein structure changes when exposed to radiation or heat. Conventional methods include techniques such as differential scanning calorimetry, fluorescence dyeing, and imaging, or spectroscopic methods such as ultraviolet absorbance and infrared. However, these pose a challenge when trying to understand the sub-micro level changes occurring within the biological tissues, whereas SV-OCT can be reliably used for measuring such minute changes in structures and can be relied upon for characterization and visualization of changes occurring during these processes. Lee, C. et al., in 2016, employed SV-OCT to demonstrate the protein denaturation and coagulation using thermal-induced egg white for experimental confirmation regarding the temperature effects as mentioned above [55]. An egg white specimen was placed on a heat plate and examined at 16 different temperatures for a homogeneous temperature distribution. The acquired 2D OCT images assessed SV both quantitatively and qualitatively, as shown in Figure 11a–d. The intensity enhancement of different regions of interest was examined, and the variance of speckles was observed as a function of temperature [55]. The quantitative parameters, such as intensity, SV, and cross-correlations, were examined as a function of temperature, and the results successfully confirmed the capability of monitoring the molecular motions of a biological specimen through the variance of speckles.

**Figure 12:**
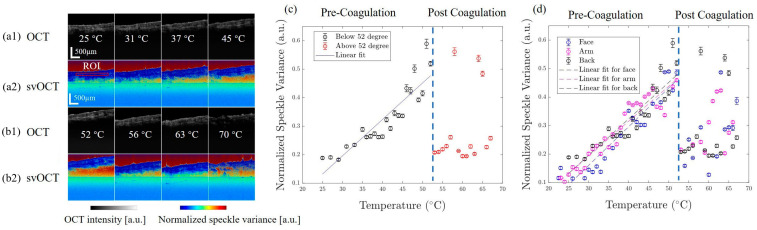
The illustration provides an overview of (**a1**,**b1**) OCT images based on average intensity and (**a2**,**b2**) their corresponding SV-OCT counterparts, capturing the “dog-ear” sample on the back throughout eight diverse heating temperatures. (**c**) Displays the linear regression relationship between normalized SV and tissue temperature before coagulation, while (**d**) demonstrates calibration outcomes on the “dog-ear” sample taken from three sites (adapted from [57]).

Cutaneous laser therapy treats port wine stains, wrinkles, and acne scars [56]. During this therapy, tissues are targeted with laser-based heating, causing minimal damage to surrounding structures. SV-OCT monitors temperature changes in ex vivo skin tissue during pulsed laser-based treatment [57]. The therapy generates heat, and SV-OCT helps observe molecular motions and SV caused by the induced heat. This assists in understanding and controlling temperature distribution during the treatment. The verifications of cutaneous laser therapy were obtained by conducting spatial and temporal temperature modeling. A normalized SV value as a function of tissue temperature is illustrated in Figure 12a,b. In contrast, the linear regressions between normalized SV and tissue temperature are depicted in Figure 12c,d. Although a rapid increase of the temperature excised in biological specimens was successfully measured in both afore-stated reports, *in vivo* assessments are required for high precision.

Since most of the SV-OCT implementations have been focused on pre-clinical research studies, translating SV-OCT into clinical settings has gained enormous interest. Ongoing research studies are underway to adapt SV-OCT systems for human imaging and clinical diagnosis. The existing challenges, such as imaging protocols, regulatory compliance, data validation, reliability, and low accuracy, must be successfully addressed to accomplish clinical translation. In clinical settings, quantitative analysis is crucial in understanding disease progression and treatment response. Thus, researchers are actively developing and refining quantitative analysis techniques for *in vivo* SV-OCT images, which can be utilized to extract quantitative parameters, such as tissue thickness, blood flow velocity, volumetric measurements, and other morphological features.

## 3. SV-OCT in Therapeutic Assessments of Various Medical Treatments

**Figure 13:**
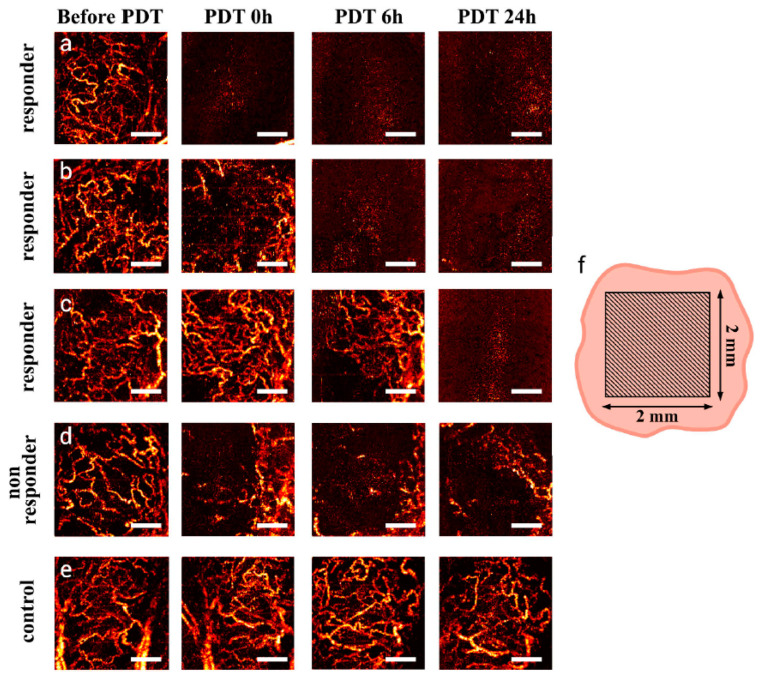
MML-OCA images depict microvascular dynamics before, immediately after, 6 h post, and a day after PDT (100 J/cm^2^, 100 mW/cm^2^). A maximum intensity projection 2D display facilitates comparison, representing 3D data to a depth of approximately 1.3 mm. Examples (**a**–**c**) show responding tumors, Example (**d**) a mildly responding tumor, and (**e**) no changes in the control. Schematic (**f**) of the scanning zone. Microvascular inhibition in responding tumors (t < 24 h) was confirmed by histology (t = 7 days). Scale bar = 500 μm on all images (adapted from [63]).

The efficacy of tumor treatments has been enhanced by applying promising targeted cancer therapy methods with lower side effects compared to conventional methods [58]. However, identifying the damages or alterations in blood vessels, blood volume, and surrounding tissues is still challenging [59]. The non-destructive and high-resolution inspection capability to assess the therapeutic effects of medical therapies is one of the most robust applications of SV-OCT. PDT is one of the frequently applied cancer treatment methods with low tissue toxicity [60,61]. However, as mentioned above, particular damages that occur in blood vessels, immune responses, and surrounding tissues are the most discussed mechanisms of PDT [62]. To examine the early microvascular PDT response-based speckle variations, M-mode-like OCT/angiography (MML-OCT/MML-OCA) was developed by Sirotkina, M.A et al. [63]. MML-OCA outperforms Doppler-OCT in flow measurements. In this MML-OCA method, bulk tissue motion artifacts resulting from tissue displacements can be significantly compensated owing to a short time lag between A-scans. As illustrated in Figure 13a–e, the monitored MML-OCA results (monitored within 6-h intervals) successfully confirmed the microvascular network and the early tumor reaction for final tumor necrosis and tumor volume reduction. In contrast, PDT-induced microvascular alteration and blood vessel injuries were further identified. The acquired results were confirmed through histology, and *in vivo*, human assessments are required before clinical application. It is worth noting that, in addition to SV-OCT, fluorescence microscopic methods have been employed for the monitoring process of PDT. However, SV-OCT is marginally superior to fluorescence microscopy due to its strong advantages, such as label-free imaging without using contrast agents, real-time monitoring capability, high-depth penetration, non-invasiveness, compatibility with other imaging modalities, and quantitative assessments. Thus, these advantages make the SV-OCT method a promising tool for assessing treatment response and optimizing PDT protocols.

**Figure 14:**
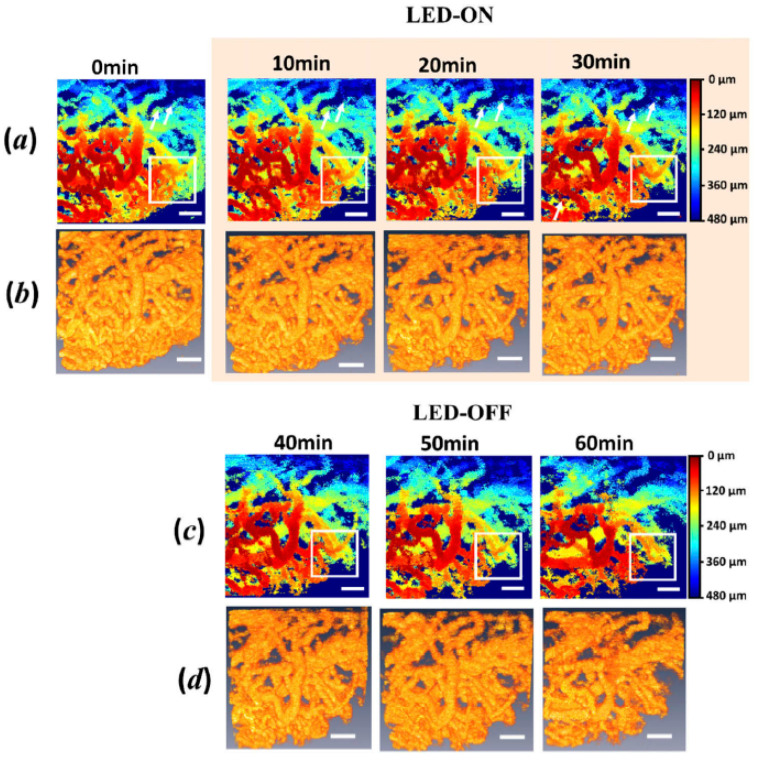
Tumor vasculature changes during and after NIR-PIT. (**a**) Illustrates the blood vessel lumen, pseudo-colored with depth (0 μm to 480 μm) during light emitting diode (LED) ON for 30 min. (**b**) Displays the corresponding 3D vessels with SV-OCT during LED ON. (**c**) Depicts the vessel lumen pseudo-colored with depth within 30 min after turning off the LED. (**d**) Shows the corresponding 3D vessels within 30 min post LED-OFF. Scale bar = 200 μm (adapted from [67]).

Near-infrared photoimmunotherapy (NIT-PIT) is another promising cancer therapy method with lower side effects [64,65]. This method is based on monoclonal antibody-photon absorber conjugate (APC) [66]. However, similar limitations to PDT can be found in NIT-PIT since identifying the effects on surrounding tissues takes time and effort. The utilization of conventional OCT along with SS-OCT-based SV-OCT for real-time visualization of the lumen in tumor blood vessels during NIR-PIT for in situ and *in vivo* specimens was reported in [67,68]. Since exposure to light-emitting diode (LED) radiation impacts tumor specimens, the SV-OCT assessments were acquired at non-LED-exposing and LED-exposing illumination stages to the specimen. The results shown in Figure 14 emphasize the vascular changes during non-exposure (Figure 14a,b) and exposure to LED radiation (Figure 14c,d). Although the locations of the tumor model were identified through the cross-sections of both OCT systems, SV-OCT was solely capable of visualizing the changes in diameter, lumen density [69], and blood volume of blood vessels during the treatment. Nevertheless, the technical functionality of the system has to be further enhanced to overcome the limitations of imaging artifacts by optimizing the repetition rate of B-scans. 

**Figure 15:**
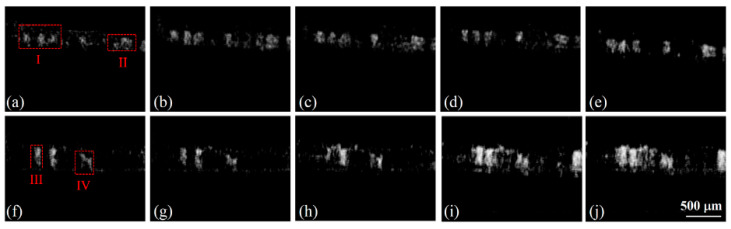
SV images derived from OCT images obtained (**a**) before FUS exposure and during exposure to different FUS powers: (**b**) 1 W, (**c**) 5 W, (**d**) 10 W, and (**e**) 15 W. Moreover, SV images derived from OCT images obtained (**f**) before FUS exposure and during exposure to various FUS powers: (**g**) 1 W, (**h**) 5 W, (**i**) 10 W, and (**j**) 15 W. Regions I and II in (**a**) are at without microbubbles, regions III and IV in (**f**) are regions with microbubbles (adapted from [23]).

**Figure 16:**
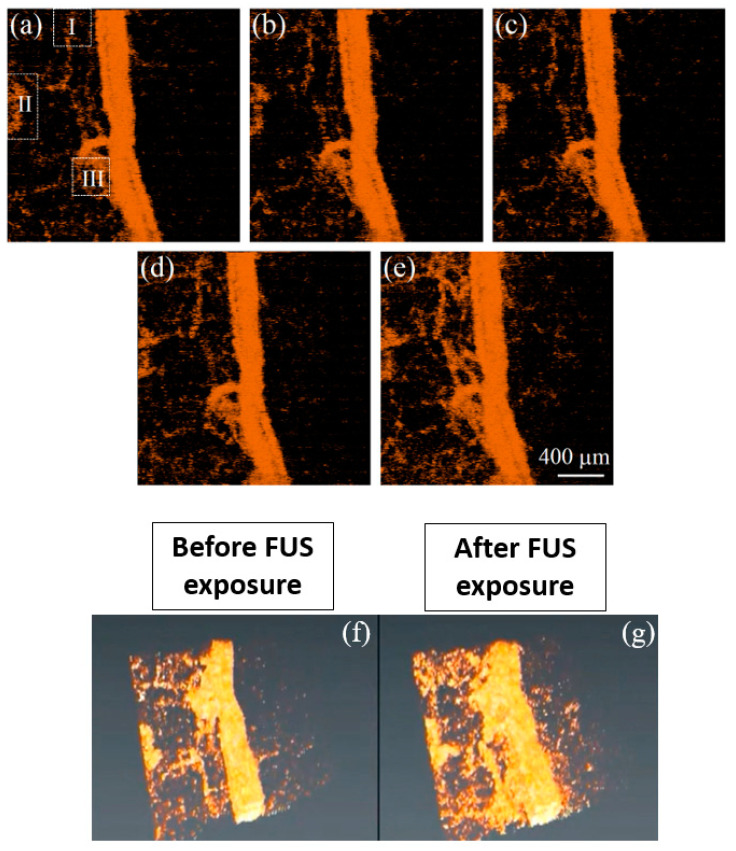
3D projection view of SV-OCT images of the mouse ear acquired (**a**) before FUS exposure and after FUS exposures of (**b**) 1 W, (**c**) 5 W, (**d**) 10 W, and (**e**) 15 W. In addition, (**f**,**g**) illustrate a comparison between 3D SV-OCT images before and after FUS exposure of 15 W. In (**a**) I, II, and III are highlighted regions to evaluate the change in the distribution of speckle variance when FUS exposure increased (partially adapted from [23]).

Moreover, the temporal vascular effects during focused ultrasound (FUS) treatment were investigated using SV-OCT by Tsai, M.T. et al. [23]. FUS is a method that increases drug delivery through blood vessels by concentrating the ultrasound energy on a target, which is applied locally and temporally, improving the vascular permeability [70]. However, non-invasively discovering the effects induced by FUS is a challenging task, which SV-OCT can sufficiently resolve. During the experimental procedure, mice were used *in vivo*, and the animals were sequentially exposed to several power limits. The results shown in Figure 15a–j and Figure 16a–g depict the SV that occurred due to the contributions from red blood cell extravasations. Figure 16 illustrates the 3D projection of a mouse ear specimen at the exposure of various power limits. Figure 15 shows that, to obtain the results of the exposures in the absence of microbubbles, regions I and II were chosen for estimating the changes in vascular areas. In contrast, regions III and IV were chosen for the case in which microbubbles were present during the FUS exposure. 

Although SV-OCT images revealed various speckles according to the induced power range, the overall results verified that the intensity and distribution of the SV are proportional to the FUS power. In addition to qualitative representation, quantitative results of SV-OCT observed the blood leakage due to the permeability enhancement induced by FUS. Here, SV-OCT was utilized to calculate SV due to blood flow and leakage, confirming the real-time applicability to assess FUS therapy. To quantitatively evaluate the change in the distribution of speckle variance, three regions depicted in Figure 16a (I, II, and III), indicated by the rectangular regions bounded by dashed lines, were chosen for estimation of the change in vascular area. 

**Figure 17:**
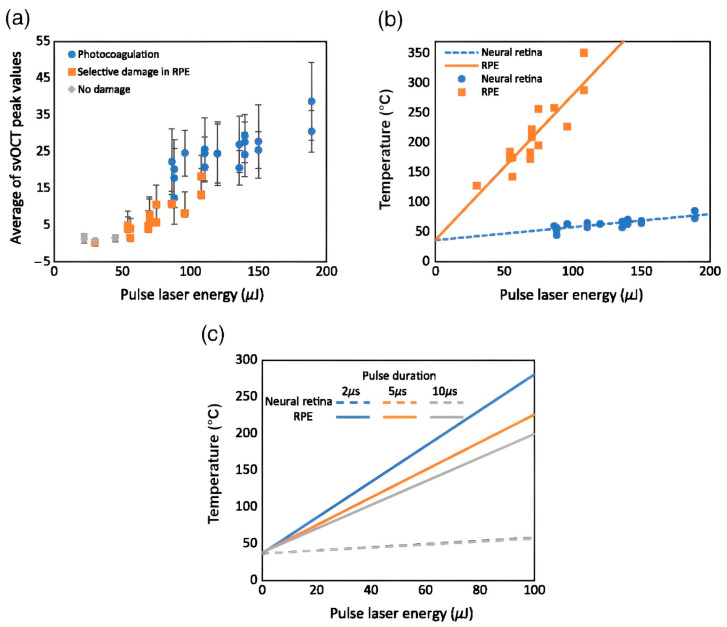
(**a**) Average peak values associated with pulse laser energy and damage range. (**b**) Shows simulated temperature profiles (lines) and estimated temperatures derived from SV-OCT intensity (shapes) at the neural retina and RPE. (**c**) Illustrates the simulated temperature at the neural retina and the RPE, varying with laser energy levels for three pulse durations—2, 5, and 10 μs (adapted from [78]).

Laser-based treatments are frequently employed in ophthalmology for various retinal diseases [57,71]. Among the therapies that are currently in practice, selective retina therapy (SRT) is one of the most effective treatment methods [72]. SRT has been frequently used to treat macula edema [73], central serous chorioretinopathy [74], and age-related macular degeneration (AMD) [75]. Currently, operating conventional ophthalmological imaging equipment has limitations in examining excessively laser-burnt regions and collateral damages [76] due to laser energy, where adjusting the laser energy is crucial. To overcome this limitation, a microsecond pulsed laser system [77] of the SRT was integrated with SV-OCT to non-invasively examine the treatment method in real time using ex vivo bovine eye specimens. The results of the Lee, S. et al. study, as shown in Figure 17a, reveal the average of SV-OCT peak values as a function of pulsed laser energy. The representative data in blue circles indicate when the laser pulses induce a lesion in the upper neural layers and in the retinal pigment epithelium (RPE) layer. At the same time, orange color squares represent the cases when the lesions were induced only on the RPE layer. Thus, the results confirmed that they successfully treated the exact locations precisely. The detailed results of the study further confirmed that SRT was successfully monitored using SV-OCT with distinctive signal variations corresponding to laser pulse irradiation.

To obtain precise assessments in ophthalmology, 2D and 3D results are essential factors [79]. Though real-time 2D SV-OCT results were feasible in most SV-OCT literature reports, acquiring 3D voxels is challenging due to the required acquisition time of volumetric variances. During the acquisition of a 3D retinal image, the subject’s movement can lead to a vague visualization of retinal vasculature [80]. Hence, to overcome this drawback, multiple volumetric composites were acquired sequentially by integrating a motion artifact elimination method [81]. To resolve the abovementioned drawback, automated image registration for motion correlation using multiple sets of SV-OCT data was utilized. This computerized image registration procedure consists of six operational steps, namely, image segmentation [82], motion detection, and image sub-division [83], Gabor filtering [84], global image registration or global placement of each strip relative to a starting reference image, local deformation of the image of the vasculature, and finally, generation of a composite image [85]. The above-processed image was further mosaiced in a wide field to obtain a visualization with a wide field of view [86]. 

**Figure 18:**
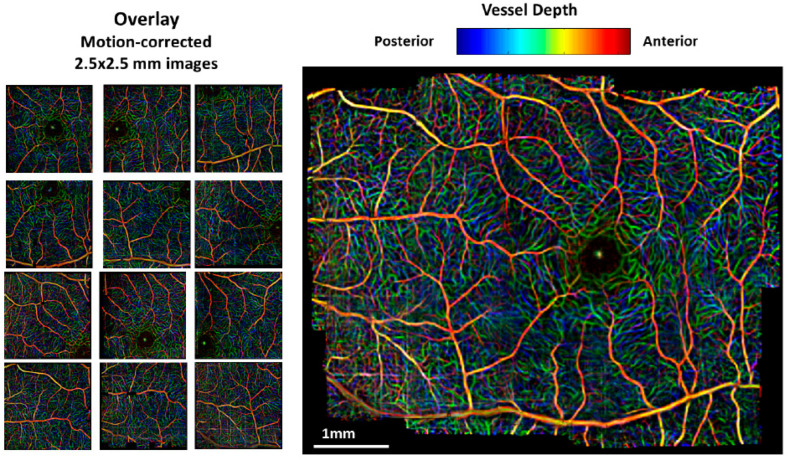
Presents a wide-field mosaic of retinal layers featuring a color-encoded depth image, incorporating information from registered mosaics of the three main vessel layers. In this representation, red denotes superficial vessels, while blue indicates deeper vessels. The individual, color-encoded, depth motion-corrected images are displayed on the left, with the nasal retina positioned on the left side and the temporal on the right within the mosaic (adapted from [15]).

Moreover, Figure 17b,c depicts simulated temperature profiles and variations at the neural retina and retinal pigment epithelium (RPE), respectively, illustrating the effects of laser energy levels on retinal temperature changes. These images were also mosaiced to facilitate wider field visualization. Figure 18 shows the acquired widefield color encoded and mosaiced depth visualizations of retinal layers. The results confirmed the ability of the developed image registration method to segment retinal data to perform layer-specific angiography using SV-OCT. 

Table 1 summarizes the key features and limitations of SV-OCT applications in medical imaging and therapy. Each application, from cardiovascular imaging to scar progression monitoring, offers unique benefits such as reduced motion artifacts, real-time tissue temperature monitoring, and non-invasive assessment of tissue changes. However, these applications also face challenges such as limited penetration depth, artifacts from temperature variations, and the need for standardized protocols. The references provide further insight into the research and development of these OCT-based techniques and highlight ongoing efforts to overcome limitations and improve their clinical utility.

The comparison mentioned above clearly demonstrates that SV-OCT holds promise as a label-free imaging technology for therapeutic assessments across a wide range of medical specialties. Ongoing SV-OCT research efforts and applications in therapeutic assessments of various medical treatments mainly focus on validating SV-OCT findings through the optimization of image analysis algorithms. Additionally, the integration of SV-OCT with other highly sophisticated and accurate imaging technologies to construct multimodal imaging devices has been another well-known fact that enhances clinical practice and treatment efficacy. 

## 4. Concluding Remarks, Future Trends, and Prospects

This comprehensive study highlights diverse applications of SV-OCT in various biomedical domains. The versatility of SV-OCT is demonstrated through pre-clinical translation and quantification capabilities for biological tissue assessment, such as effectiveness in monitoring scar progression, examining rodent spinal cord vasculature, and assessing temperature effects on tissues. The usage of SV-OCT for therapeutic assessments has been well-proven through tumor treatments, cutaneous laser therapy, and focused ultrasound treatments. Moreover, SV-OCT proves versatility in ophthalmology, enabling real-time examination of laser-based treatments and providing essential insights into retinal vasculature. To further compare the previously reported methods in a contrasting manner, Table 1 illustrates a summary of key features and limitations of SV-OCT incorporated methods in biomedical applications, showcasing the potential non-invasive and high-resolution imaging merits with significant applications across different fields, paving the way for future biomedical research and clinical practices. Each application, from cardiovascular imaging to scar progression monitoring, offers unique benefits such as reduced motion artifacts, real-time tissue temperature monitoring, and non-invasive assessment of tissue changes. However, these applications also face challenges such as limited penetration depth, artifacts from temperature variations, and the need for standardized protocols. The references provide further insight into the research and development of these OCT-based techniques and highlight ongoing efforts to overcome limitations and improve their clinical utility.

Exploring the future capabilities of SV-OCT reveals novel paths for advancement that could reshape various domains beyond current applications. In healthcare, democratized access is one of the focal points, emphasizing miniaturization for affordability and portability to everyone. Furthermore, the rapid assessment of skin lesions, wound healing, or ophthalmic conditions directly at the point of care facilitates timely diagnosis and treatment, while transmission of this vital medical information via a reliable communication network enables physicians to assess patients in remote conditions through telemedicine applications. Also, the high-resolution images of SV-OCT reveal deep tissue structures, which hold potential significance in monitoring various neurological disorders like multiple sclerosis, Alzheimer’s disease, or Parkinson’s disease. This label-free imaging capability allows the visualization of alterations in brain tissue morphology, tracking disease advancement, and evaluating the effectiveness of neuroprotective therapies. Moreover, SV-OCT might advance the development of brain–computer interfaces through its detailed imaging of neural tissue and connectivity patterns. In the meantime, pushing the resolution limits of SV-OCT uncovers promising significance for future progress. Therefore, exploring novel light sources, such as supercontinuum lasers or ultrafast lasers with adaptive optics, could enable deeper tissue penetration and higher-resolution imaging. Moreover, SV-OCT can be improved by adding different multimodal imaging methods, such as photoacoustic and hyperspectral imaging. This synergy provides comprehensive information while unlocking novel paths for diagnosis. Among the applications, measuring body functions such as blood flow velocity, oxygen saturation, or metabolic activity in real-time through functional imaging could enhance disease characterization. In addition, developing targeted probes or contrast agents specifically for SV-OCT imaging could enable visualization of specific biomarkers and early detection of diseases at the molecular level. Moreover, the high resolution of SV-OCT can play a significant role in developing new brain–computer interfaces for neurological applications and rehabilitation.

In the modern era dominated by artificial intelligence (AI), the integration of SV-OCT with advanced technologies such as machine learning (ML) and deep learning (DL) presents a significant prospect across various applications. AI enhances various aspects of SV-OCT, including image acquisition, processing, analysis, and interpretation. It improves image quality by reducing noise and automating tasks such as the segmentation of structures and the detection of abnormalities. Using ML algorithms to reconstruct high-resolution images from under-sampled data can significantly reduce acquisition times and improve the signal-to-noise ratio. Through extensive training on large SV OCT datasets, DL algorithms can accurately identify and classify lesions, enhancing diagnostic efficiency. It can be used to obtain real-time feedback, helping surgeons with precise interventions and better outcomes. Also, DL algorithms can be used to monitor the progression of new skin conditions, providing valuable information for enhancing strategies. Those advanced technologies can personalize medicine by examining specific patient information and SV-OCT images and tailoring treatment plans according to unique disease characteristics and therapy responses. In conclusion, the potential of the future of SV-OCT is vast. Research and development promise revolutionary advancements, ultimately improving healthcare outcomes globally.

## Figures and Tables

**Table 1:** Summary of key features and limitations of SV-OCT incorporated methods in biomedical applications.

Application	Key Features	Limitations	References
Mouse Cardiovascular Imaging	Reduced Cardio-Respiratory Motion Simplified Stabilization Jig Depth Resolved Imaging High Microvascular Network Resolution	Limited Depth Penetration Bulk Motion Sensitivity The trade-off with Transverse Resolution Need of post-processing	[22,30,41,50,87]
Temperature Effects on Tissues	Non-invasive detailed imaging of tissues under varying temperature conditions Monitoring protein denaturation and coagulation in real-time Facilitating precise quantitative measurements of temperature effects Consistent trends across different sites	Limited penetration depth for deeper tissue assessment. *In vivo* assessments are crucial for achieving high precision. The potential presence of artifacts, attributed to temperature variations. The challenge in post-coagulation temperature monitoring.	[55,57]
Photodynamic Therapy (PDT)	Potential advantages of SV-OCT over fluorescence microscopy Detection of thrombosis, a key microvascular reaction to PDT Confirms microvascular network changes and early tumor reactions through histology	Artifacts from Multiple Scattering Influence of Interframe Bulk Tissue Motion Emphasizes the need for *in vivo* human assessments before clinical application	[20,60,61,62,63,88]
Near-Infrared Photoimmunotherapy (NIT-PIT):	Visualizes vascular changes during NIT-PIT Monitors changes in vessel diameter, lumen density, and blood volume during treatment Contrast Agent-Free Imaging	The technical functionality of the system needs enhancement Imaging Speed Limitations Limited FOV and Spatial Resolution	[66,67,68]
Focused Ultrasound Treatment	Non-invasive tissue heating and ablation Investigates temporal vascular effects during FUS treatment Utilizes SV-OCT for real-time assessment of FUS therapy Quantitative Comparison with and without Microbubbles 2D and 3D imaging, providing a comprehensive view of the vascular changes Speckle Variance for Blood Leakage Detection	SV-OCT images reveal various speckles but limited quantitative results. Limited FOV and Spatial Resolution Resolution Trade-offs and Image Artifacts Data Processing Complexity Cost Considerations Lack of standardized protocols for combining OCT with FUS	[23,70]
Laser-Based Treatments in Ophthalmology	Linear Dependence of SV-OCT Signal on Laser Energy Ultrahigh-resolution imaging allows for better visualization and segmentation of individual intraretinal layers Phantom Study Validation Two radiation modes, classic and ramping, are explored Real-time feedback from SV-OCT imaging	Melanin Concentration Variability Handling and interpreting the large volume of data generated by SV-OCT Validation is needed through clinical studies and trials	[57,71]
Scar Progression Monitoring	Non-invasive assessment of scar progression longitudinal monitoring of scar progression Sensitive to various tissue changes induced by laser treatments Quantitative assessment of scar progression	Artifact Mitigation Variability in optical properties of ocular tissues Lack of Standardization of Protocols	[31,51,52,53,56]

## Data Availability

No data were used for the research described in the article.
